# Nucleoside Analogs and Perylene Derivatives Modulate Phase Separation of SARS-CoV-2 N Protein and Genomic RNA In Vitro

**DOI:** 10.3390/ijms232315281

**Published:** 2022-12-03

**Authors:** Julia Svetlova, Ekaterina Knizhnik, Valentin Manuvera, Vyacheslav Severov, Dmitriy Shirokov, Ekaterina Grafskaia, Pavel Bobrovsky, Elena Matyugina, Anastasia Khandazhinskaya, Liubov Kozlovskaya, Nataliya Miropolskaya, Andrey Aralov, Yuri Khodarovich, Vladimir Tsvetkov, Sergey Kochetkov, Vassili Lazarev, Anna Varizhuk

**Affiliations:** 1 Federal Research and Clinical Center of Physical-Chemical Medicine of Federal Medical Biological Agency, 119435 Moscow, Russia; 2 Department of Molecular and Translational Medicine, Moscow Institute of Physics and Technology, 141701 Dolgoprudny, Russia; 3 K.I. Skryabin Moscow State Academy of Veterinary Medicine and Biotechnology, 109472 Moscow, Russia; 4 Engelhardt Institute of Molecular Biology, Russian Academy of Science, 119991 Moscow, Russia; 5 Chumakov Federal Scientific Center for Research, Development of Immune-and-Biological Products of Russian Academy of Sciences, 108819 Moscow, Russia; 6 Institute of Molecular Genetics, National Research Centre ‘Kurchatov Institute’, 123182 Moscow, Russia; 7 Shemyakin-Ovchinnikov Institute of Bioorganic Chemistry, RAS, 117997 Moscow, Russia; 8 Research and Educational Resource Center for Cellular Technologies, The Peoples’ Friendship University of Russia, 117198 Moscow, Russia; 9 Institute of Biodesign and Complex System Modeling, I.M. Sechenov First Moscow State Medical University, 119991 Moscow, Russia

**Keywords:** phase separation, nucleocapsid protein, RNA, SARS-CoV-2, small-molecule antivirals

## Abstract

The life cycle of severe acute respiratory syndrome coronavirus 2 includes several steps that are supposedly mediated by liquid–liquid phase separation (LLPS) of the viral nucleocapsid protein (N) and genomic RNA. To facilitate the rational design of LLPS-targeting therapeutics, we modeled N-RNA biomolecular condensates in vitro and analyzed their sensitivity to several small-molecule antivirals. The model condensates were obtained and visualized under physiological conditions using an optimized RNA sequence enriched with N-binding motifs. The antivirals were selected based on their presumed ability to compete with RNA for specific N sites or interfere with non-specific pi–pi/cation–pi interactions. The set of antivirals included fleximers, 5′-norcarbocyclic nucleoside analogs, and perylene-harboring nucleoside analogs as well as non-nucleoside amphiphilic and hydrophobic perylene derivatives. Most of these antivirals enhanced the formation of N-RNA condensates. Hydrophobic perylene derivatives and 5′-norcarbocyclic derivatives caused up to 50-fold and 15-fold enhancement, respectively. Molecular modeling data argue that hydrophobic compounds do not hamper specific N-RNA interactions and may promote non-specific ones. These findings shed light on the determinants of potent small-molecule modulators of viral LLPS.

## 1. Introduction

Despite the recent progress in managing coronavirus infectious disease 2019 (COVID-19) and the largely successful vaccination programs, severe acute respiratory syndrome coronavirus 2 (SARS-CoV-2) remains a global threat, and the development of anti-SARS-CoV-2 therapeutics is ongoing. With regards to new druggable targets, increasing attention is being paid to intracellular liquid–liquid phase separation (LLPS) of the viral nucleocapsid protein (N) and genomic RNA (gRNA). Growing evidence supports the importance of N-gRNA biomolecular condensates (droplets) formed in the host cell cytoplasm [1] for the life cycle of SARS-CoV-2 [2]. In particular, they are implicated in replication [3], immune evasion [4], and virion packaging [5]. Justifying the search for specific modulators of the LLPS-mediated steps of the SARS-CoV-2 cycle, the unique molecular grammar of N-gRNA condensates has recently been uncovered [6,7]. It comprises key elements of N and gRNA primary and secondary structures (“stickers”) that define transient heterotypic (N-RNA) or homotypic (N-N) interactions within droplets, as well as droplet plasticity-defining elements (“spacers”) in-between stickers. 

**Figure 1:**
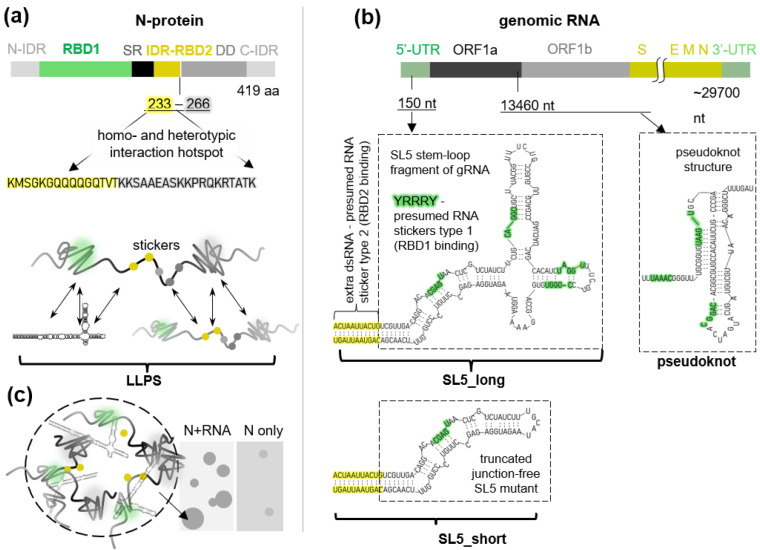
Schematic representation of the SARS-CoV-2 N protein (**a**) and gRNA fragments (**b**) used to model SARS-CoV-2 biomolecular condensates (**c**). Presumed RNA secondary structures are shown and presumed interaction hotspots (RNA stickers) are marked.

N protein contains two structured domains, namely the N-terminal RNA binding domain (RBD1) and the C-terminal dimerization domain (DD), flanked by three intrinsically disordered regions (IDRs) (Figure 1a). As was evidenced by the cross-linking mass spectrometry assays, the Ser/Arg-rich (SR) region-adjoining RNA-binding part of the central IDR (RBD2) and the neighboring part of the DD form a particularly large number of homo/heterotypic contacts upon N-gRNA condensation [4,5]. They contain multiple Arg and Lys residues (presumed stickers) interspersed with non-charged amino acids (presumed spacers) and are recognized as essential for LLPS [4,5]. In viral gRNA, two types of stickers have been identified recently. These are dsRNA stretches and YRRRY motifs, where R is a purine nucleotide, and Y is a pyrimidine nucleotide [7,8] (Figure 1b). 

The dsRNA stretches are enriched in the ORF1ab-harboring 5′-part of SARS-CoV-2 gRNA and can interact with N RBD2. Such interactions supposedly disrupt N dimers and trigger LLPS by exposing the hydrophobic surface of the DD [7]. The presumed LLPS-mediated clustering of N at the 5′-terminus of gRNA may repress ORF1ab translation and thus facilitate the generation and translation of subgenomic RNAs (sgRNAs) that encode structural SARS-CoV-2 proteins. Among them, N-sgRNA is the most abundant, presumably due to the presence of several YRRRY stickers within its ORF-preceding transcription-regulatory sequence (TRS) “body”. The YRRRY motifs, found in the regulatory sequences of all sgRNAs, are recognized by N RBD1. The juxtaposition of a TRS “body” and a 5′ TRS “leader”, typically followed by the leader–body fusion upon discontinuous transcription [9], may be facilitated by N dimerization or condensation. The condensation must eventually initiate viral packaging, likely through the maturation of multiple droplets into regularly spaced ribonucleocapsid packaging “eggs” (RNPs) consistent with a cryo-electron tomography-based “birds-nest” model of the inside of the virion [10]. Finally, clustering of N at both 5′ and 3′ termini of gRNA may trigger gRNA cyclization, ensuring the correct packaging stoichiometry (a single gRNA copy per virion). 

The spectrum of N-mediated processes suggests the importance of timely N accumulation and phase separation. Interference with N-RNA phase behavior is expected to repress RNA replication and virion assembly (e.g., by hindering the synthesis of structural proteins or RNP nucleation/maturation). Perhaps the best-known native modulator of SARS-CoV-2 LLPS is ATP. Prevalent in host cells, it controls multiple phase transitions in norm and pathology [11]. Due to a combination of hydrophobic and negatively charged fragments, it can either solubilize N monomers (e.g., via masking the hydrophobic DD fragment [12] or intra-IDR stickers) or, conversely, promote separation (e.g., via the ligand-bridging mechanism [13]), depending on the ATP:N ratio [14]. We assume that the LLPS-modulating properties of ATP may be mimicked to some extent by antiviral nucleoside/nucleotide analogs. Surprisingly, such compounds have received little (if any) attention in reported LLPS modulation assays [4,8,15,16]. 

In this study, we investigated the LLPS-modulating potential of the recently reported nucleoside/nucleotide-based antivirals. Our ultimate goal was to clarify whether the interference of such small molecules with N-gRNA separation may contribute to the inhibition of SARS-CoV-2 replication. We also aimed to elucidate the general features of potent LLPS modulators. For that, we compared nucleoside/nucleotide-based antivirals containing hydrophobic and polar/negatively charged fragments with perylene-based antivirals containing hydrophobic (polyaromatic) and polar/positively charged fragments. To test the propensity of these compounds for modulating N-gRNA separation, we obtained a simple in vitro model of SARS-CoV-2 condensates (Figure 1c) based on the current understanding of the N-gRNA interaction grammar. 

## 2. Results and Discussion

### 2.1. N Protein Phase Separates with gRNA Fragments That Contain Sterically Accessible Stickers

**Figure 2:**
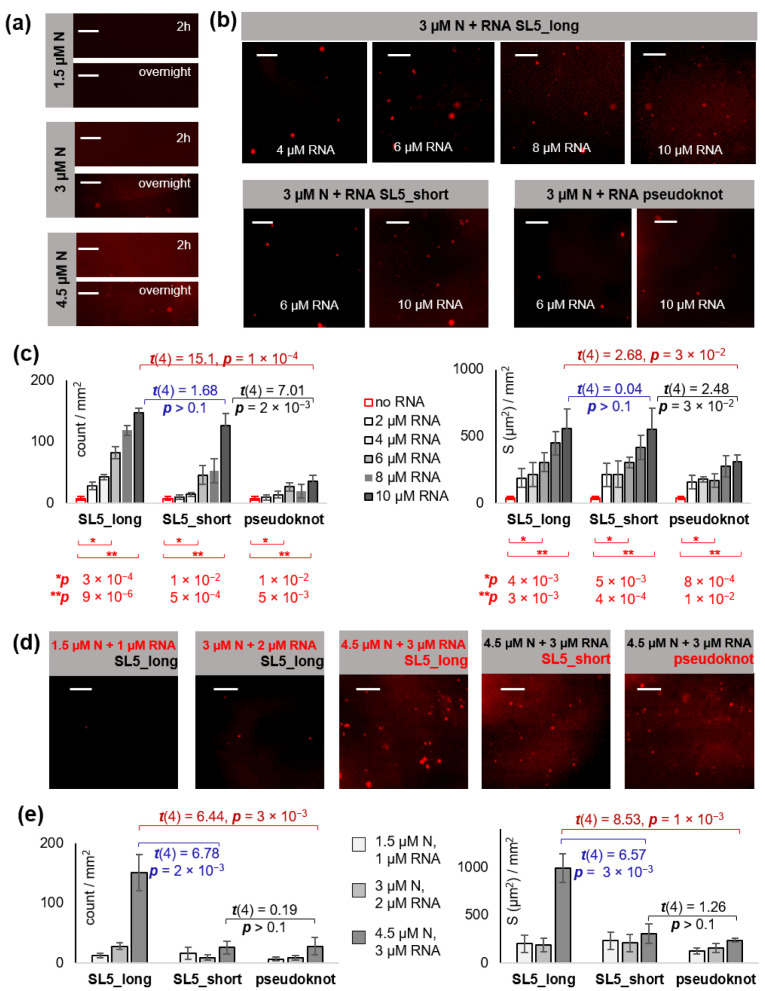
N-RNA phase separation under pseudo-physiological conditions: dependence on RNA structure and concentration. (**a**) N separation in the absence of RNA. Fluorescence microscopy images were obtained after incubation of RED-labeled N (1.5–4.5 µM) in the working buffer (20 mM Tris-HCl buffer, pH 7.4) at 37 °C for 2 h or overnight. (**b**) RNA effects on the separation of 3 µM N. The images of N mixtures with increasing RNA concentrations were obtained after an overnight incubation in the working buffer at 37 °C. (**c**) Summary of RNA effects on the separation of 3 µM N. Left: Average number of droplets (count) per mm^2^ coverslip. Right: average total droplet area (S, µm^2^) per mm^2^ coverslip. (**d**) RNA effects in 1.5:1 N:RNA mixtures. The images of the mixtures with increasing total concentrations were obtained after overnight incubation in the working buffer at 37 °C. (**e**) Summary of the separation of 1.5:1 N:RNA mixtures. Left: average droplet number (count) per mm^2^ coverslip. Right: average total droplet area (S, µm^2^) per mm^2^ coverslip. Scale bars: 10 µm.

We aimed to obtain N-RNA droplets that would be a reasonably adequate model of the intracellular condensates [3,4] (i.e., sustain physiological conditions) for subsequent screening of LLPS modulators. The protein was expressed in bacteria to avoid posttranslational modifications because they are mostly absent in native N at early infection stages, although Ser residues in the SR site may eventually undergo LLPS-limiting phosphorylation [17]. Consistently with a previous report [6], zero to marginal LLPS was detected for 1.5–4.5 µM N protein, labeled with a red light-emitting dye (RED), under physiological conditions (pH 7.5, 150 mM NaCl, 37 °C) in the absence of RNA. First signs of LLPS (7 ± 3 droplets per mm^2^ coverslip with a total area equal to 38 ± 10 µm^2^) appeared at N concentration equal to 3 µM after overnight incubation (Figure 2a), and the apparent partitioning coefficient (PC = F_droplet_/F_solution_) was equal to 9 ± 6. For subsequent experiments with RNA, we selected N concentration equal to 3 µM, because it has been used in previous studies of N-gRNA LLPS [7], is close to the previously reported saturation concentration detected in a cell-free system with non-viral RNA [6], and can be achieved in infected cells rapidly after virion unpackaging [6,18]. 

The RNA sequences were selected based on the abundance of double-stranded YRRRY motifs (sticker type 1) [7,8]. We used a modified fragment (SL5_long) of the 5′-UTR of SARS-CoV-2 gRNA. This fragment is a part of the N-protein-binding principal site 1 and has been predicted to form a branched stem loop SL5 [8]. The modification, i.e., the complementary flanks that extend the SL5 stem (sticker type 2), has been used previously to enhance the LLPS-promoting behavior of SL5 [8]. To ascertain whether sticker abundance or the steric factor determines N recruitment and phase separation, we additionally analyzed a truncated YRRRY-poor non-branched SL5 mutant (SL5_short) and the interlocked sticker-rich pseudoknot structure. The pseudoknot is located near the slippery site at the ORF1a/ORF1b boundary and promotes programmed ribosomal-1 frameshifting [19] (Figure 1b). The RNA sequences were obtained by in vitro transcription, and their secondary structures were confirmed by circular dichroism spectroscopy (Figure S1).

First, we fixed N concentration and added increasing concentrations of SL5_long, SL5_short, or pseudoknot RNA (Figure 2b,c). The apparent PC value remained nearly constant at RNA concentrations up to 6 µM and was equal to 10 ± 3 in the presence of SL5_long or SL5_short and 6 ± 2 in the presence of the pseudoknot RNA. The average number of N droplets increased 11-fold, 6-fold, and 4-fold upon the addition of 6 µM SL5_long, SL5_short, and the pseudoknot RNA, respectively. The total droplet area increased 8-fold (SL5_long and SL5-short) and 4-fold (pseudoknot RNA). These changes were statistically significant and indicated LLPS enhancement. Increasing the RNA concentration to 10 µM reduced PC ~2-fold, suggesting the droplets became looser, although their number and their total area increased (Figure 2). SL5_long and SL5_short appeared to be equally potent LLPS facilitators and were superior to the pseudoknot RNA.

Next, we focused on SL5_long and SL5_short and performed an additional series of experiments to verify their relative potency at N:RNA ratio equal to 1.5:1 (Figure 2d,e). We used an excess of N in order to disclose potential weak RNA stickers, such as stem junctions or loops. We fixed the N:RNA ratio and varied the overall concentration of both components. At a relatively high concentration (4.5 µM N and 3 µM RNA), SL5_long was superior to SL5_short, which supports the assumption about weak stickers in SL5_long (Figure 2e). Despite the presence of multiple presumed stickers, the pseudoknot RNA was the least potent LLPS facilitator in most cases (Figure 2d,e), perhaps because the access to its stickers is hindered due to their interlocking. We conclude that the phase separation of N protein under physiological conditions is promoted by gRNA fragments with sterically accessible stickers. 

For further analysis, we selected the SL5_long:N ratio equal to 2:1 (6 µM RNA and 3 µM N) because it ensured noticeable droplet formation, while PC remained equal to 10 ± 3. This N:RNA ratio (1 N protein molecule per ~80 nt) is lower than that used previously (~3 N molecules per nt [7]) but very close to that expected in cells (1 N per ~70 nt, assuming a single RNA copy and 38 RNPs, ~12 N molecules each, per virion [10]).

**Figure 3:**
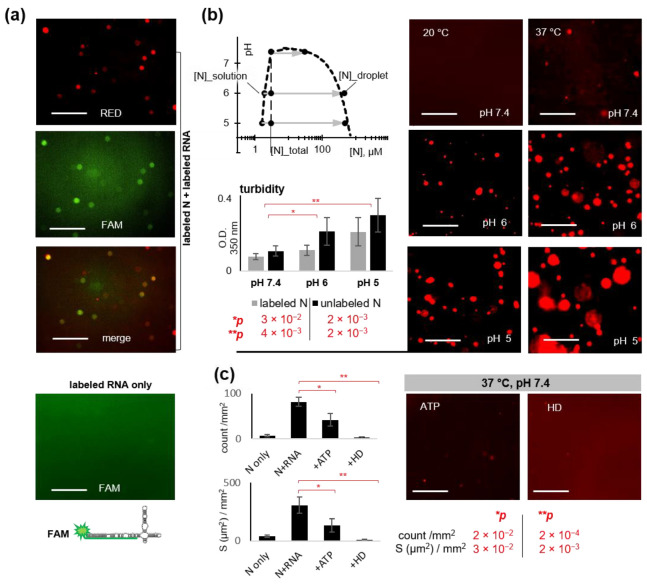
Condition-dependence of the N-RNA condensates and their sensitivity to known LLPS modulators. (**a**) Verification of N colocalization with RNA. Representative fluorescence microscopy images of the mixtures of RED-labeled N (3 µM) and FAM-labeled tr-SL5_long/SL5-tag (6 µM) after overnight incubation in 20 mM Tris-HCl, pH 7.4, at 37 °C. (**b**) Temperature and pH-dependence of the N-RNA condensates (3 µM N and 6 µM SL5_long). Left top panel: schematic phase diagram summarizing the microscopy-based analysis of droplet formation in the labeled N-RNA mixtures after 2 h of incubation in 20 mM Tris-HCl, pH 7.4, or 20-mM sodium-acetate, pH 5–6, at 37 °C. Left bottom diagram: summary of the turbidity assays with the labeled and unlabeled N-RNA mixtures under the same conditions. Right panel: representative fluorescence microscopy images of the labeled N-RNA mixtures after 2 h of incubation at pH 5–7.4 at 20 °C and 37 °C. (**c**) Sensitivity of the N-RNA condensates (3 µM N and 6 µM SL5_long) to known LLPS modulators ATP and 1,6-hexanediol (HD) at 37 °C. Scale bars: 10 µm.

Because SL5_long proved to be the most potent LLPS facilitator, we used it in all subsequent experiments. To confirm the inclusion of SL5_long into the condensates, we obtained its analog with a FAM-labeled stem (tr-SL5_long/SL5-tag) and mixed it 2:1 with N. Following the overnight incubation at 37 °C, droplets similar to those detected in SL5_long:N mixtures (Figure 2b) were observed in both red (N) and green (RNA) channels (Figure 3a). In the absence of N, tr-SL5_long/SL5-tag showed no apparent phase separation (Figure 3a). The incomplete colocalization of red and green spots may be attributed to droplet floating. However, we cannot exclude minor artifacts related to aberrant tr-SL5-long/SL5-tag folding, considering the slightly reduced CD amplitude of tr-SL5-long/SL5-tag compared to SL5_long (Figure S1). Anyway, we conclude that SL5_long is likely included into N condensates to some extent.

### 2.2. Electrostatic N-RNA Interactions May Facilitate Hydrophobicity-Dependent N Separation

According to a recent hypothesis, the effects of RNA on N separation can be explained within the framework of the conventional surface tension-related hydrophobicity-driven LLPS concept [20]. Briefly, transient N-N interactions are presumably promoted by the exposure of the hydrophobic part of DD upon its partial denaturation. The denaturation can be caused by electrostatic interactions between RNA and the positively charged stickers at the IDR/DD boundary (e.g., Arg or Lys residues in RBD2) [7]. To verify this hypothesis and additionally characterize the condensates, we investigated their condition-dependence and sensitivity to known modulators of hydrophobic and electrostatic interactions. 

First, we verified the temperature dependence of N-RNA condensates. The exposure of the hydrophobic DD core must be favored by its thermal denaturation [7]. Because few condensates were observed in the 4.5 µM N solution under physiological conditions even in the absence of RNA (Figure 2a), low critical solution temperature (LCST) must be below the physiological value. At room temperature (~20 °C), no droplets were found even in the presence of RNA, suggesting 20 °C < LCST < 37 °C (Figure 3b). 

Next, we analyzed the effects of salt concentration and pH. Both homotypic (N-N) and heterotypic (N-RNA) interactions must be dependent on the ionic strength of the solution. Low ionic strength supposedly facilitates contacts between the negatively charged RNA backbone and the positively charged N stickers. At the same time, it may enhance RNA-RNA repulsion and weaken all hydrophobic interactions. We observed no apparent LLPS at low (10 mM) salt concentrations; the fluorescence microscopy images of the N-RNA mixture were similar to those obtained in the absence of RNA (Figure 2a). This result supports the hydrophobicity-driven separation. 

At a physiological salt concentration (150 mM) and pH values in the range of 5–7.4, N-RNA mixtures showed increasing LLPS with decreasing pH. In the weakly acidic media, the condensates became apparent even after a relatively short (2 h) incubation at both 20 °C and 37 °C (Figure 3b). Incubation overnight gave similar results, except for the minor droplet flocculation, which agrees with previous observations [21,22]. This trend, revealed by fluorescence microscopy, was additionally confirmed by turbidity assays (Figure 3b). We attribute the effect of pH to partial deprotonation of the positively charged N protein, which increases the surface tension and promotes the hydrophobicity-driven separation. 

Finally, we verified the effects of the commonly used LLPS modulators 1,6-hexanediol (HD) and ATP. Condensates held together by hydrophobic interactions are typically sensitive to HD [23]. Under physiological conditions, 10% HD caused nearly total droplet dissolution in the N-RNA mixtures (Figure 3c). This result agrees with previous reports [8]. Both electrostatic and hydrophobic interaction-dependent condensates are sensitive to ATP [11]. Under physiological conditions, we observed a substantial (~50%) reduction in the total droplet number and area in the presence of 20 µM ATP (Figure 3c). This concentration is well below the reported Kd value of ATP-RBD1 complex (3.3 ± 0.4 mM) [14] and approximately two orders of magnitude lower than the average intracellular concentration of ATP [24]. However, the ATP:N ratio (~7:1) is comparable to that expected locally at early infection steps, when ATP supposedly facilitates nucleocapsid unpacking [14].

To summarize this part, N-SL5_long condensates appear to be a reasonably accurate simplified model of the viral condensates because they show appropriate dependence on the external conditions and sensitivity to the known modulators. The condensates are likely held together by both electrostatic and hydrophobic interactions, but hydrophobicity appears to play the key role.

### 2.3. Nucleoside Analogs and Perylene Derivatives Modulate N-RNA LLPS In Vitro

**Figure 4:**
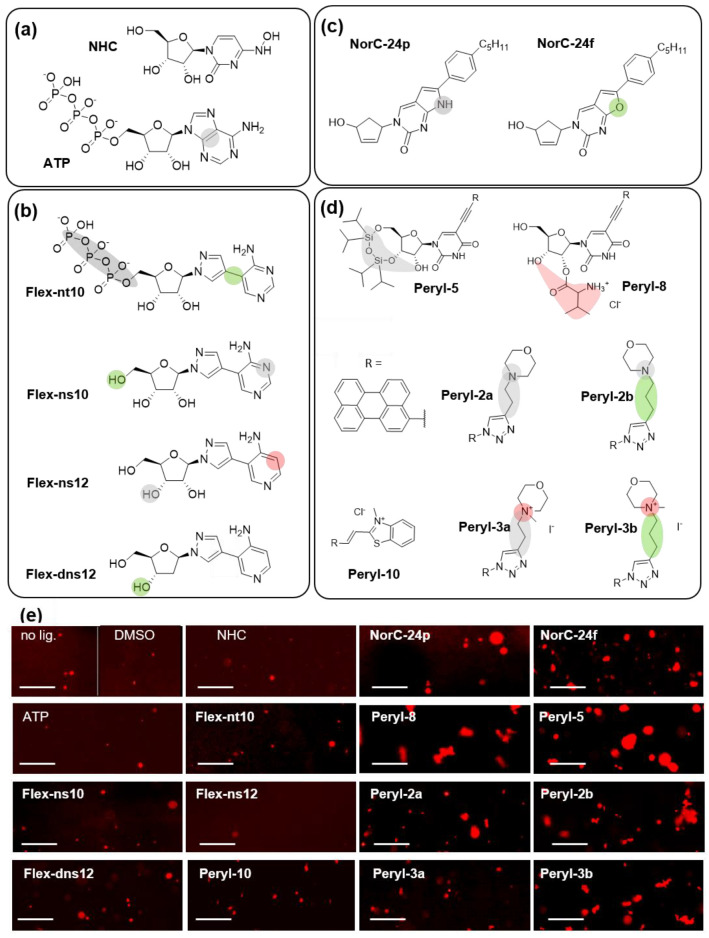
LLPS-modulating nucleoside analogs and perylene derivatives. (**a**) Control small molecules: N^4^-hydroxycitidine (NHC), control nucleoside-like antiviral; adenosine triphosphate (ATP), control LLPS modulator. (**b**) Fleximer (Flex) analogs of ATP, adenosine, and deoxyadenosine. (**c**) 5′-Norcarbocyclic analogs of pyrimidine nucleosides. (**d**) Uridine analogs with perylene (Peryl) substituents in the nucleic base and non-nucleoside perylene derivatives. In each subset of the small molecules, modified residues are highlighted (grey). Those enhancing or inhibiting the formation of N-RNA condensates are marked with green or red, respectively. (**e**) Fluorescence microscopy images of the N-RNA mixtures (3 µM N and 6 µM SL5_long) after overnight incubation with 20 µM small molecule in 20 mM Tris-Cl, pH 7.4, at 37 °C. Scale bars: 10 µm.

The proposed model of the SARS-CoV-2 N-RNA condensates was used to search for LLPS modulators among known antiviral agents and their derivatives (Table 1). Inspired by ATP, we focused on nucleoside analogs (Figure 4a–c) and used ATP as a control modulator (Figure 4a). Considering the presumed role of hydrophobic interactions and cation-pi as well as pi–pi contacts in N-RNA separation, we also included condensed aromatic small molecules (perylene derivatives) into the set of the tested compounds (Figure 4d). 

Fleximers (Figure 4b) are purine nucleic base/nucleoside mimetics in which the six- and five-membered heterocycles are connected by a C–C bond, enabling their rotation to promote “induced fit”-type binding with a target protein [25,26]. Initially designed as molecular probes for studying RNA/DNA-recognizing enzymes, fleximers have shown significant promise in antiviral research, including the development of coronavirus inhibitors [27]. Among the available fleximers, we selected the ATP mimetic Flex-nt10 (analog of 8-aza-7-deazaadenosine 5'-triphosphate), the adenosine mimetics Flex-ns10 (8-aza-7-deazaadenosine analog) and Flex-ns12 (8-aza-3,7-dideazaadenosine analog), and the deoxyadenosine mimetic Flex-dns12 [28,29].

In addition to purine nucleoside derivatives, we considered a well-known cytidine analog NHC (β-D-N^4^-hydroxycytidine, Figure 4a) [30] and several recently reported pyrimidine derivatives (Figure 4c,d) that inhibited SARS-CoV-2 in vitro. These derivatives included 5′-norcarbocyclic (NorC) analogs of 3H-pirrolo[2,3-d]-pyrimidine-2-one (NorC-24p) and 3H-furano[2,3-d]-pyrimidine-2-one (NorC-24f) nucleosides with a hydrophobic 4-pentylphenyl substituent (Figure 4c) [29] and perylene-harboring uridine analogs Peryl-8 and Peryl-5 (neutral and positively charged derivatives, respectively, Figure 4d) [31]. To verify the importance of charged and hydrophobic fragments, we also tested the non-nucleoside neutral perylene derivatives Peryl-2a and Peryl-2b, their positively charged analogs Peryl-3a and Peryl-3b, and an additional positively charged compound with in vivo-confirmed antiviral activity Peryl-10 (Figure 4d) [31]. The codes of all small molecules were taken from the previous works [29,31] and the prefixes indicate small molecule types.

Effects of the small molecules on phase separation of N-RNA mixtures (3 µM N and 6 µM SL5_long) under physiological conditions (pH 7.4, 37 °C) were analyzed using fluorescent microscopy imaging (Figures S1–S3), and the changes in the average droplet number (count) or total droplet area (S) were calculated (Table 1). At concentrations ≥40 µM, some of the small molecules formed insoluble aggregates or induced substantial droplet flocculation (Figure S4), which hampered quantitative characterization, so for comparative analysis we tested all small molecules at a concentration of 20 µM (Figure 4e).

Under the selected conditions, S did not exceed 5% of the coverslip area in most cases, and the apparent partitioning coefficient (PC = F_droplet_/F_solution_ = 10 ± 4) changed insignificantly (within error) with increasing droplet number (count). S showed a clear linear correlation with droplet count in the cases of ATP, NHC, NorC, and Flex nucleoside derivatives (R^2^ = 0.83). The correlation was less apparent for non-nucleoside perylene derivatives (R^2^ = 0.63), probably because they promoted the formation of larger droplets (i.e., facilitated droplet coalescence). Within each small-molecule series (Flex, NorC, or Peryl), droplet size distribution was broad but constant. Thus, we selected S as a key indicator of LLPS efficiency.

Fleximers turned out to be mildly efficient LLPS modulators, comparable to ATP and NHC. Flex-ns12 suppressed N-RNA separation, while other derivatives enhanced it to a moderate extent (2–3-fold). The 5′-norcarbocyclic nucleoside derivatives NorC-24p and NorC-24f were robust facilitators of LLPS. They increased average S values ~11-fold, and ~15-fold, respectively. Top facilitators of LLPS were found in the Peryl series: the hydrophobic non-nucleoside derivative Peryl-2a increased S ~25-fold, and the nucleoside analog Peryl-5 increased it ~50-fold.

The hydrophobic perylene derivatives were more potent than amphiphiles, and the derivatives with an extended flexible linker (propyl) between the polyaromatic system and the morpholino ring (Peryl-2b and Peryl-3b) were superior to their shorter-linker (ethyl) analogs (Peryl-2a and Peryl-3a). Importantly, all perylene derivatives induced a more or less pronounced N-RNA gelation and the formation of irregular-shaped aggregates. This occurred during droplet maturation and involved RNA, which we confirmed using tr-SL5_long/SL5tag (Figure S2). In contrast to perylene derivatives, droplets obtained in the presence of fleximers or NorC nucleoside analogs maintained a spherical shape. For clear visualization, we investigated them under LLPS-enhancing non-physiological conditions (pH 6 and pH 5) and observed similar trends (Figure S3). Interestingly, at pH 7.4 droplet formation was already noticeable after 2 h of incubation in all cases (Figures S2 and S5). Further incubation (overnight) resulted in increased droplet area, while the apparent number of droplets remained approximately constant or even decreased slightly (Figures S2 and S5). This observation suggests rapid droplet formation and subsequent gradual coalescence.

To summarize this part, NorC nucleoside analogs and hydrophobic perylene derivatives proved to be top modulators of N-RNA separation. Both of these groups of small molecules enhanced LLPS.

**Table 1:** LLPS modulation assays with known small-molecule antiviral agents and their analogs.

Code	Effects on the N-RNA Condensates ^a^		Antiviral Activity, IC50 ± SD, µM ^b^
	S/S_control_ ± SD	Count/Count_control_ ± SD	
ATP	0.4 ± 0.1	0.5 ± 0.2	-
NHC	0.5 ± 0.3	0.4 ± 0.1	8 ± 5, refs. [29,31] ^c^
NorC-24p	11 ± 1	6.2 ± 0.8	21 ± 6, ref. [29] ^c^
NorC-24f	15 ± 1	9 ± 2	≥50 ^c^
Flex-nt10^b^	2 ± 1	4.7 ± 0.3	-
Flex-ns10	2.9 ± 0.9	2.4 ± 0.4	>100 ^c^
Flex-ns12	0.4 ± 0.1	0.4 ± 0.1	>100 ^c^
Flex-dns12	3 ± 1	4 ± 1	>100 ^c^
Peryl-8	23 ± 7	1.4 ± 0.4	1.3 ± 0.4, ref. [31] ^c^
Peryl-5	50 ± 10	1.5 ± 0.4	>100, ref. [31]
Peryl-2a	17 ± 6	1.8 ± 0.3	11 ± 2, ref. [31]
Peryl-2b	25 ± 8	2.5 ± 0.7	>100, ref. [31]
Peryl-3a	6 ± 2	1.3 ± 0.3	1.9 ± 0.9, ref. [31]
Peryl-3b	21 ± 7	5 ± 1	9 ± 4, ref. [31]
Peryl-10	4 ± 1	0.6 ± 0.3	1.5 ± 0.9, ref. [31]

^a^ Effects of the small molecules (20 µM) on N-RNA separation evidenced by fluorescence microscopy imaging are summarized in two parameters: the normalized average number of droplets (count/count_control_) and their normalized total area (S/S_control_), where control is buffer for water-soluble compounds (ATP and Flex-nt10) and 10% DMSO for others. ^b^ Similarly to ATP, Flex-nt10 does not cross the cellular membrane, hence no IC50 data. ^c^ Fleximer derivatives were tested for antiviral activity following the protocol published in [29,31]. Top Peryl [31] and NorC [29] antivirals and NHC were used as positive controls.

### 2.4. LLPS Modulation Might Underlie Antiviral Effects of NorC Nucleoside Analogs but Does Not Correlate with the Effects of Other Tested Small Molecules

Screening of nucleoside analogs and perylene derivatives provided insufficient data for identifying characteristic features of potent LLPS inhibitors but revealed a possible general motif of LLPS enhancers, namely a nucleoside(-like) moiety with an aromatic substituent. Although previously discussed LLPS-related strategies for antiviral drug design rely on LLPS inhibitors [4,8,15], enhancers might also restrict viral replication, e.g., by hampering gRNA unpacking or inducing aberrant/premature RNP assembly. We questioned whether the small-molecule-induced LLPS changes show any covariance with IC50 values.

As evident from Table 1, the extent of N-RNA droplet alteration by perylene derivatives does not covary with the reported antiviral effects in VERO cells. Thus, mechanisms unrelated to phase separation—e.g., the disturbance of the viral envelope membrane and the prevention of membrane fusion by perylene derivatives [32,33]—must prevail over LLPS modulation. Similarly, LLPS modulation by NHC, if present, must be non-decisive in vivo. NHC is phosphorylated in cells and is incorporated into the gRNA chain upon viral replication to eventually inhibit SARS-CoV-2 through lethal mutagenesis [30].

Fleximers can also undergo phosphorylation in cells and may trigger mutagenesis or inhibit RNA-dependent RNA polymerase (RdRp). Their activity against SARS-CoV-2 has not been verified so far. We tested Flex-nt10 (an ATP analog) for RdRp inhibition using a previously developed assay [34] (Supplementary Text Box S1 and Figure S6) and observed relatively weak activity. The apparent EC50 (half-maximal effective concentration) value was ≥300 µM in the presence of 10 µM ATP. We also tested the effects of Flex-nt10 and fleximer nucleoside analogs on viral replication in VERO cells using a visual cytopathic effect assay following the previously published protocol [29,31]. Top NorC and Peryl derivatives were used as positive controls. Their effects agreed with previous reports [29,31], while fleximer derivatives showed no significant activity at concentrations ≤ 100 µM (Table 1). Thus, moderate LLPS modulation by fleximer derivatives, if present in cells, must be insufficient to prevent SARS-CoV-2 replication.

In contrast to mutagens like NHC, close nucleoside mimics, sterically hindered replication terminators, or other RdRp substrates [35], 5′-norcarbocyclic derivatives cannot be phosphorylated in cells and are not incorporated into the growing RNA chain [29], so the lethal mutagenesis mechanism can be excluded. Nevertheless, the NorC derivatives inhibited SARS-CoV-2 replication in VERO cells (Table 1). NorC-24p (IC50 = 21 ± 6 [29]) appeared superior to NorC-24f, though these compounds showed significant and profound cytotoxicity, respectively, at a concentration of 100 µM, which prevented accurate IC50 calculation. In previous studies, NorC-24p has shown toxicity to VERO and human lung adenocarcinoma cells with CC50 values close to 50 µM but was non-toxic to human fibroblasts at 100 µM [29,36]. In the LLPS modulation assay, NorC-24p was active at a concentration of 20 µM, which is within the antiviral activity range (Table 1). At concentrations ≤10 µM (Figure S7), it showed minor to zero activity. Because the LLPS modulation data are roughly in agreement with the activity in VERO cells, and no other mechanisms of action have been proposed for NorC-24p so far, we hypothesize that its anti-SARS-CoV-2 effect might be at least partly due to the interference with N-RNA separation.

**Figure 5:**
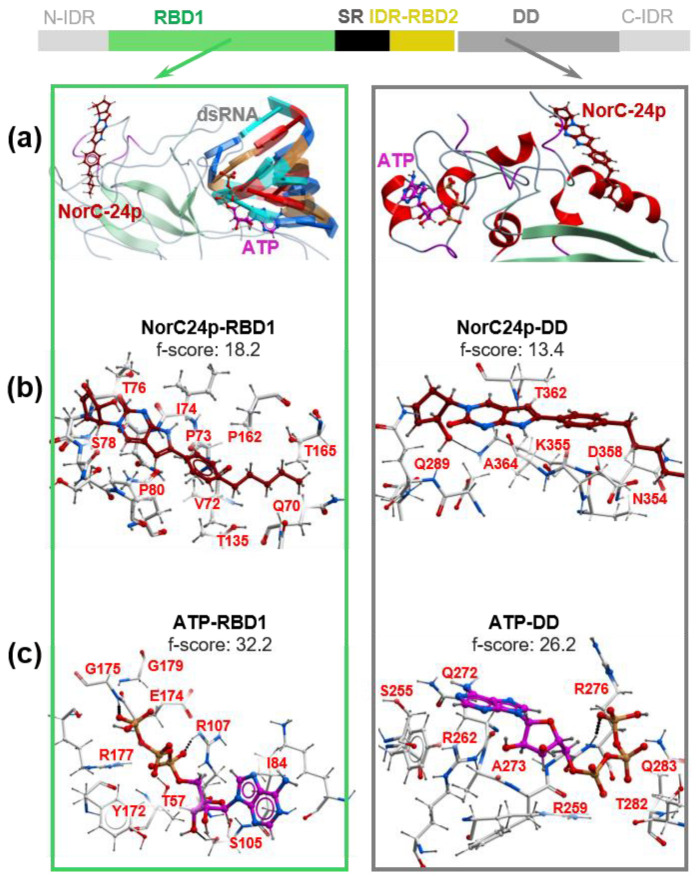
Molecular modeling of NorC-24p and ATP interactions with N protein. (**a**) Key binding sites revealed by docking of NorC-24p and ATP to N-protein RBD1 and DD. (**b**) Best-scoring conformations of NorC-24p complexes with N and respective scoring functions. (**c**) Best-scoring conformations of ATP complexes with N and respective scoring functions.

Finally, to clarify the opposite effects of NorC-24p (phase separation enhancer) and the control molecule ATP (phase separation inhibitor) in the LLPS modulation assays, we modeled interactions of these compounds with N protein in silico. NorC-24p and ATP were docked to N domains that reportedly form multiple homotypic (N-N) or heterotypic (N-RNA) contacts upon phase separation (Figure 1a):

- the N-terminal RNA binding domain (RBD1, PDB ID: 7ACS), which supposedly accounts for the specific heterotypic interactions [7];
- the C-terminal dimerization domain (DD, PDB ID: 6YUN), which accounts for specific homotypic and various non-specific interactions [5];

Docking revealed different binding sites of ATP and NorC-24p in each N fragment (Figure 5). A comparison of the scoring functions points to RBD1 as the most likely binding site for both ATP and NorC-24p. In RBD1, ATP blocked the dsRNA-recognizing facet between the nonpolar/uncharged polar (I84, S106, and T57) and positively charged (R107 and R177) amino acid residues. NorC-24p targeted the opposite facet of RBD1 between the amino acid residues Q70-P80 and appeared unlikely to interfere with RNA-RBD1 contacts. It occupied the hydrophobic cleft on the RBD1 surface without blocking potential stickers, while its aromatic residue remained accessible for presumed cation–pi or pi–pi interactions. In DD, ATP and NorC-24p contacted distinct alpha helices (amino acids Q272-Q283 and N354-A364, respectively), and the NorC-24p-occupated site was slightly closer to the dimerization interphase. To summarize, ATP shielded some of the stickers in N RBDs, while NorC-24p introduced extra stickers (Figure 5). These findings are consistent with ATP and NorC-24 behavior in LLPS modulation assays.

## 3. Conclusions

Using recombinant N-protein (N) and the YRRRY-rich fragment of the SARS-CoV-2 genome with the extended dsRNA stretch (Figure 1), we obtained biomolecular condensates that imitate the intracellular viral condensates. They sustained physiological pH, ionic strength, and temperature (Figure 2). They showed condition dependence and sensitivity to known modulators that were consistent with the current views on the molecular grammar of SARS-CoV-2 N-RNA LLPS (Figure 3).

Because N-RNA LLPS is repressed by HD and the excess of ATP according to our data (Figure 3) and previous reports, we searched for LLPS modulators among other nucleoside-based and hydrophobic/amphiphilic small molecules that can be considered for antiviral drug development (Figure 4). The majority of the tested small molecules enhanced biocondensate formation in vitro (Table 1), suggesting they might disrupt the timing of intracellular RNP assembly and other LLPS-mediated processes. The effects were particularly pronounced in the cases of perylene derivatives with uncharged polar substituents and 5′-norcarbocyclic nucleoside derivatives.

The lead perylene derivative Peryl-5 increased condensate formation ~50-fold in our model system and can be used as a robust LLPS modulator in other cell-free systems. However, in cells, it likely targets lipid membranes rather than N-RNA condensates. In contrast, LLPS modulation by 5′-norcarbocyclic derivatives may partly account for their antiviral activity. In silico analysis of the 5′-norcarbocyclic derivative NorC-24p (Figure 5) suggests that it does not hamper specific N-RNA interactions and might facilitate transient non-specific interactions. We conclude that perylene derivatives, such as Peryl-5, are helpful facilitators of LLPS in model systems, while 5′-norcarbocyclic nucleoside derivatives hold promise for targeting viral condensates in cells. However, in view of the substantial toxicity of the 5′-norcarbocyclic nucleoside derivatives, comprehensive studies of their interactions with stress granules and other membraneless organelles of the host cells are needed.

## 4. Materials and Methods

### 4.1. Nucleocapsid Protein Expression and Labeling

RNA of the SARS-CoV-2 Wuhan variant was isolated from a COVID-positive donor’s sample using TRIzol LS Reagent (Thermo-Fisher Scientific, Madison, WI, USA), and reverse transcription of the full-length N protein-encoding fragment was performed using the RevertAid RT Reverse Transcription Kit (Thermo Fisher). The cDNA was PCR-amplified and purified by preparative agarose gel electrophoresis, then digested with BamHI and SalI endonucleases and ligated into the modified (i.e., lacking the signal peptide sequence) pET22b plasmid that was digested with the same enzymes. The correct assembly of the resulting plasmid pET-CoV2-gN(H), encoding C-His6-tagged N protein, was confirmed by Sanger sequencing.

*E. coli* BL21-gold(DE3) cells were transformed with the pET-CoV2-gN(H) plasmid. A single colony was inoculated into 50 mL of LB medium containing ampicillin (150 lg/mL) and grown in a shaking incubator for 8 h at 30 °C. Following the incubation, the culture was inoculated into 1 L of the fresh LB medium (100 lg/mL ampicillin) and cultivated in a shaker-incubator at 37 °C to an OD600 of ~0.8. Expression was induced by adding IPTG to a final concentration of 0.5 mM. The cells were cultured for an additional 4 h at 37 °C. After incubation, the cells were spun down and disrupted by sonication. The lysate was clarified by centrifugation and filtration. Recombinant C-His6-tagged N-protein was purified by metal chelate affinity chromatography (purity: ≥90%) and labeled (1:1) with a far-red-emitting RED dye using the RED-NHS 2nd Generation Protein Labeling Kit (Nanotemper).

### 4.2. In Vitro Transcription and Circular Dichroism Spectroscopy

Three hairpin-forming modified fragments of SARS-CoV-2 gRNA, Wuhan variant, were obtained by in vitro transcription from T7 promoter-containing dsDNA templates that had been assembled from synthetic oligonucleotides and confirmed by Sanger sequencing. SL5_long [7] is a YRRRY-motif-rich branched stem-loop fragment of gRNA flanked by artificial complementary sequences that form a dsRNA stretch. Its mutant SL5_short is a truncated non-branched stem-loop with a single YRRRY motif. Pseudoknot is a native YRRRY-rich interlocked stem-loop gRNA fragment. The sequences of respective DNA templates are provided below. The T7 promoter is in bold font. The artificial dsDNA stretch is in italics, and the YRRRY motifs are underlined.

SL5-long (dsDNA template, sense strand):

**TAATACGACTCACTATAG**GGAGA*ACTAATTACTG*TCGTTGACAGGACACGAGTAACTCGTCTATCTTCTGCAGGCTGCTTACGGTTTCGTCCGTGTTGCAGCCGATCATCAGCACATCTAGGTTTCGTCCGGGTGTGACCGAAAGGTAAGATGGAGAGCCTTGTCCCTGGTTTCAACGA*CAGTAATTAGT*

SL5-short (dsDNA template, sense strand):

**TAATACGACTCACTATAG**GGAGA*ACTAATTACTG*TCGTTGACAGGACACGAGTAACTCGTCTATCTTTGCATAAGATGGAGAGCCTTGTCCCTGGTTTCAACGA*CAGTAATTAGT*

Pseudoknot (dsDNA template, sense strand):

**TAATACGACTCACTATAG**GGAGAGTTTTTAAACGGGTTTGCGGTGTAAGTGCAGCCCGTCTTACACCGTGCGGCACAGGCACTAGTACTGATGTCGTATACAGGGCTTTTGAT

Reverse transcription was performed using a 2 μg DNA template and HiScribe™ T7 High Yield RNA Synthesis Kit (New England Biolabs, Ipswich, MA, USA) following the manufacturer’s protocol. Then, the reaction mixture was diluted with a DNAse I buffer and treated with RNAse-free DNAse I at 37 °C to remove the template. RNA was precipitated from cold ethanol, and its purity was verified by electrophoresis in 2% agarose.

FAM label was introduced at the 3′-RNA terminus via the hybrid DNA-RNA duplex tag. For that, a truncated version of the target hairpin (tr-SL5_long) was obtained as described above and hybridized with the synthetic FAM-labeled DNA complement (SL5tag). The resulting duplex has substantial thermal stability (predicted T_m_ > 60 °C) to sustain physiological conditions.

tr-SL5_long (dsDNA template, sense strand):

**TAATACGACTCACTATAG**GGAGA*ACTAATTACTG*TCGTTGACAGGACACGAGTAACTCGTCTATCTTCTGCAGGCTGCTTACGGTTTCGTCCGTGTTGCAGCCGATCATCAGCACATCTAGGTTTCGTCCGGGTGTGACCGAAAGGT

SL5-tag (ssDNA):

AAGATGGAGAGCCTTGTCCCTGGTTTCAACGA*CAGTAATTAGT*-FAM

All RNA and RNA/DNA concentrations were measured using NanoDrop 2000 (Thermo Fisher). Secondary structures of the SL5_long, tr-SL5_long/SL5-tag, SL5_short, and pseudoknot RNA were verified by circular dichroism (CD) spectroscopy. The CD spectra of 0.7 µM RNA solutions in the working buffer (20 mM Tris-HCl, pH 7.4, and 150 mM NaCl) were registered at room temperature using a Chirascan spectrophotometer (Applied Photophysics) and a 1 cm optical path quartz cuvette. To visualize the difference between folded (partially folded) and unfolded (mostly unfolded) RNA, we additionally analyzed SL5_long at 80 °C. To select an appropriate annealing procedure, we compared CD spectra of SL5_long after slow annealing, which supposedly yields the thermodynamically favorable structure but allows for the undesired intermolecular folding, and rapid annealing, which supposedly facilitates intramolecular folding. Rapid annealing was performed as follows: the sample was incubated at 90 °C for 5 min and then snap-cooled on ice. Slow annealing was performed as follows: the sample was incubated at 90 °C for 5 min and then cooled gradually (over 2.5–3 h) to room temperature. For tr-SL5_long/SL5-tag, we also considered a two-step procedure. First, tr-SL5_long was annealed rapidly to enable stem-loop formation. Then, SL5-tag was added, and the mixture was incubated for 2 h at +4 °C to enable duplex formation. The final annealing procedures were selected for each RNA based on the CD data. Because the highest amplitude of the A-form-specific CD band (Figure S1) was obtained using rapid (SL5_long) and sequential (tr-SL5_long/SL5-tag) annealing, these procedures were used in all subsequent experiments prior to mixing RNA with N for LLPS modulation assays.

### 4.3. Fluorescent Microscopy Imaging, Turbidimetry, and Statistical Analysis

To obtain N-RNA condensates, fluorescently labeled recombinant N-protein (3–9 μM) was mixed with RNA (1–10 μM) in the RNAse-free 20 mM Tris-HCl buffer, pH 7.4, or the 20 mM sodium acetate buffer, pH 5 or 6 (all buffers were supplemented with 150 mM NaCl) and incubated at 20 °C or 37 °C for 2 h or overnight prior to fluorescent microscopy imaging or turbidimetry assays. To evaluate the effects of the small-molecule antivirals on the formation of the condensates, stock solutions of the antivirals in the working buffer or DMSO were added to freshly prepared N-RNA mixtures to a final concentration of 5–20 μM (the final DMSO concentration was 10%).

For turbidimetry assays, all mixtures were prepared in 384-well plates (Perkin Elmer, Waltham, MA, USA), and optical density at 350–600 nm was registered using M200 Tecan plate reader (Tecan Group Ltd., Männedorf, Switzerland).

For fluorescence microscopy imaging, 3 μL of the N-RNA mixture was placed between glass slides and analyzed using a Nikon Eclipse Ti2 microscope (Nikon, Tokyo, Japan). The droplets were inspected visually for floating. To bona fide discriminate liquid condensates from flocculating aggregates and solid particles, several images of the same area were taken with a time lag of several seconds and compared.

Using the ImageJ 1.53a software, we calculated the partitioning coefficient (PC = F_droplet_/F_solution_ = C_droplet_/C_solution_, where F is fluorescence intensity and C is concentration) and the average droplet area normalized by the coverslip area (S_droplet_/S_total_). N distribution between droplets and solution was estimated based on Equation (1), which is valid provided the total amount of the protein (C_total_·V_total_) remains constant (no precipitation):

C_total_·V_total_ = C_solution_·V_total_·(1 − V_droplet_/V_total_) + PC·C_solution_·V_droplet_, (1)

Provided all droplets have a spherical shape, volume ratio can be recalculated to area ratio, and Equation (1) can be rewritten as follows:

C_solution_ = C_total_/[1 − (S_droplet_/S_total_)^1.5^ + PC·(S_droplet_/S_total_)^1.5^] (2)

C_droplet_ = C_solution_·PC (3)

All experiments were performed in two independent repeats, and in each case at least three large-field (square millimeter) scans were analyzed. Data are presented as means ± SD. The significance of the difference of the mean droplet numbers or S values or between two samples was verified using a standard two-tailed Student’s test.

### 4.4. SARS-CoV-2 Inhibition Tests

The inhibitory activity of nucleoside analogs and the control compound (NHC) against the SARS-CoV-2 strain PIK35 was verified as was described previously [29,31,37]. Briefly, the compound-virus mixtures were added to the confluent Vero cell monolayers. The cells were incubated for 5 days, and then the cytopathic effect was evaluated by microscopy imaging.

### 4.5. Molecular Modeling

3D Models of the ATP and NorC-24 were built using ICM-Pro 3.9 2 [38]. Partial charges on all atoms were calculated as previously described [39]. Briefly, the conformational space of the small molecules was scanned in ICM-Pro 3.9-2 using a molecular-mechanical approach, a Monte Carlo method, and the mmff force field [40]. The minimal conformation was further optimized to identify the geometry of the lowest energy, and the electron density distribution was calculated by a density functional theory method DFT/M06-2X/6-311 + g(d,p) with implicit consideration of the solvent effect with application of the conductor-like polarizable continuum model (CPCM). Then, the Merz-Singh-Kollman scheme [41] was applied to the electron density distribution to calculate the grid for the electrostatic potential fitting with the following parameters: (6/41 = 10)—the number of surfaces around the atoms and (6/42 = 17)—the density of test points on these surfaces. Finally, the restrained electrostatic potential method [42] was applied for the calculation of the partial atomic charges. All quantum mechanics simulations were carried out using the Gaussian 09 program (https://gaussian.com/, accessed on 2 December 2022).

3D models of the N RNA-binding domain, dimerization domain, and the partly disordered central region (aa 233–266) were taken from PDB (IDs: 7ACS, 6YUN, and 7PKU, respectively). Docking was performed using ICM-Pro 3.8.6. The small molecules were rendered flexible, and the protein was fixed. Prior to the docking procedure, the structures of the protein and the small molecules were converted into an ICM object. The parameters needed for interatomic energy calculation and the partial charges for the atoms of the target were taken from the force field ECEPP [43]. The biased probability Monte Carlo minimization procedure [44] was used for global energy optimization. The best conformations were selected based on the energy scoring function as reported by Abagyan and Totrov [45].

## Data Availability

The data presented in this study are available in the article and supplementary material.

## Supplementary Materials

The following supporting information can be downloaded at: https://www.mdpi.com/article/10.3390/ijms232315281/s1, Figure S1: CD spectra of the RNA samples, Figures S2–S5: additional fluorescence microscopy images of the N-RNA condensates obtained in the presence of perylene derivatives, 5′-norcarbocyclic nucleoside analogs, and fleximer nucleoside analogs; Text Box S1: details on the RdRP inhibition assay; Figure S6: respective gel scans, and Figure S7: summary of the concentration dependence of nucleoside analogs in LLPS assays.
