# Supplementary material for: Nucleoside Analogs and Perylene Derivatives Modulate Phase Separation of SARS-CoV-2 N Protein and Genomic RNA In Vitro
Source: Int J Mol Sci. 2022 Dec 3;23(23):15281. doi: 10.3390/ijms232315281 (PMC9738865; doi:10.3390/ijms232315281)
Supplement: Supplementary file 1 [file ijms-23-15281-s001.zip › ijms-2020163-supplementary.pdf]

Nucleoside analogs and perylene derivatives modulate phase separation of SARS-CoV-2 N protein and genomic RNA in vitro

J. Svetlova, E. Knizhnik, V. Manuvera, V. Severov, D. Shirokov, E. Grafskaia, P. Bobrovsky, E. Matyugina, A. Khandazhinskaya, L. Kozlovskaya, N. Miropolskaya, A. Aralov, Yu. Khodarovich, V. Tsvetkov, S. Kochetkov, V. Lazarev, A. Varizhuk*

*Address correspondence to annavarizhuk@rcpcm.org

SUPPORTING INFORMATION

Figure S1. Verification of RNA secondary structures by circular dichroism (CD) spectroscopy______ 2

Figure S2. LLPS modulation assays with perylene derivatives_________________________________ 3

Figure S3. LLPS modulation assays with 5′-norcarbocyclic and fleximer nucleoside derivatives_____ 4

Figure S4. LLPS and other phase transitions at high concentrations of the small molecules_________ 5

Figure S5. Droplet maturation assays with 5′-norcarbocyclic nucleoside derivatives_______________ 8

Text box S1. SARS-CoV-2 RdRp inhibition assay: in vitro transcription___________________________ 5

Figure S6. Inhibition of SARS-CoV-2 RdRp by Flex-nt10 in competitive assays___________________ 6

Figure S7. Concentration dependence of the effects of nucleoside analogs on N-RNA separation____ 7

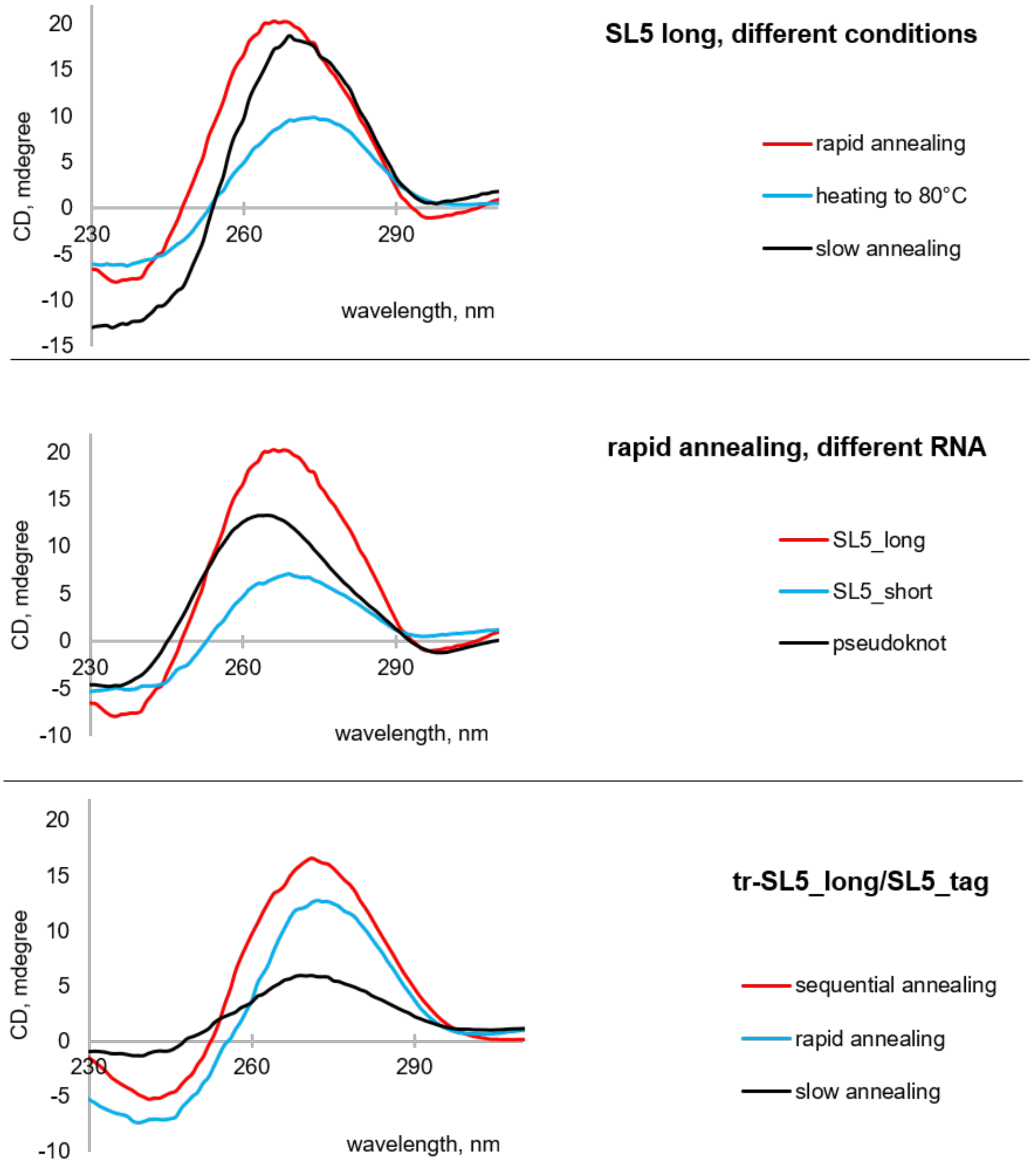

Figure S1. Verification of RNA secondary structures by circular dichroism (CD) spectroscopy. All CD spectra were obtained using 0.7 µM RNA solutions in the working buffer (pH 7.4) at room temperature unless otherwise mentioned. Rapid annealing: the sample was incubated at 90°C for 5 min and then snap-cooled on ice. Slow annealing: the sample was incubated at 90°C for 5 min and then cooled gradually to room temperature. Sequential annealing of tr-SL5_long/SL5_tag: the RNA strand (tr-SL5_long) was annealed rapidly, then the complementary DNA strand (SL5_tag) was added, and the mixture was stored for 2 h at +4°C.

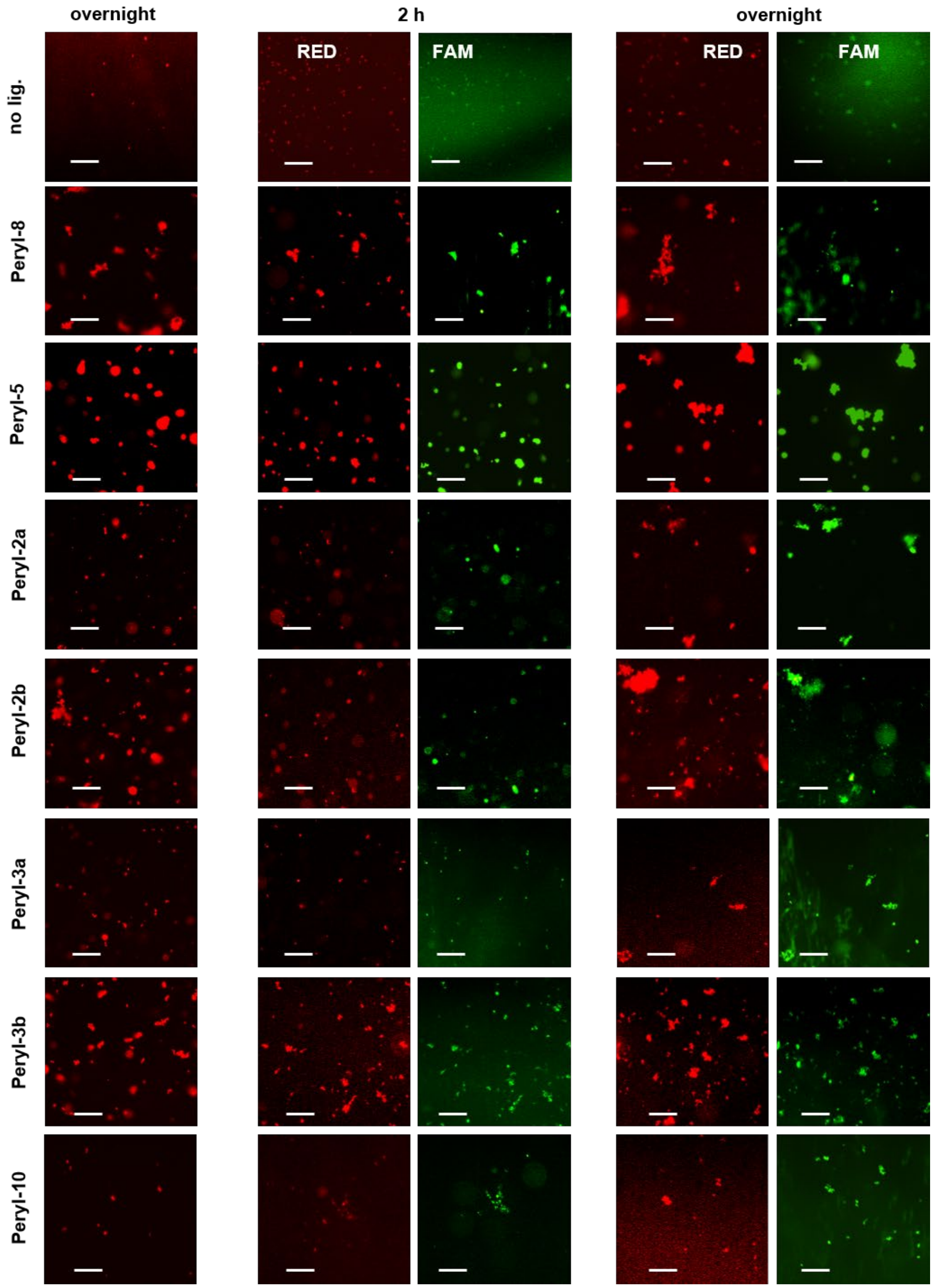

Figure S2. LLPS modulation assays with perylene derivatives. Left: fluorescence microscopy images of the mixtures of RED-labeled N (3 µM) and SL5_long (6 µM) obtained after overnight incubation with 20 µM small molecule in the working buffer (pH 7.4) at 37°C. Middle and right panels: similar assays with FAM-labeled tr-SL5_long/tagSL5 instead of SL5_long (incubation for 2 h and overnight, respectively). Scale bars: 10 µm.

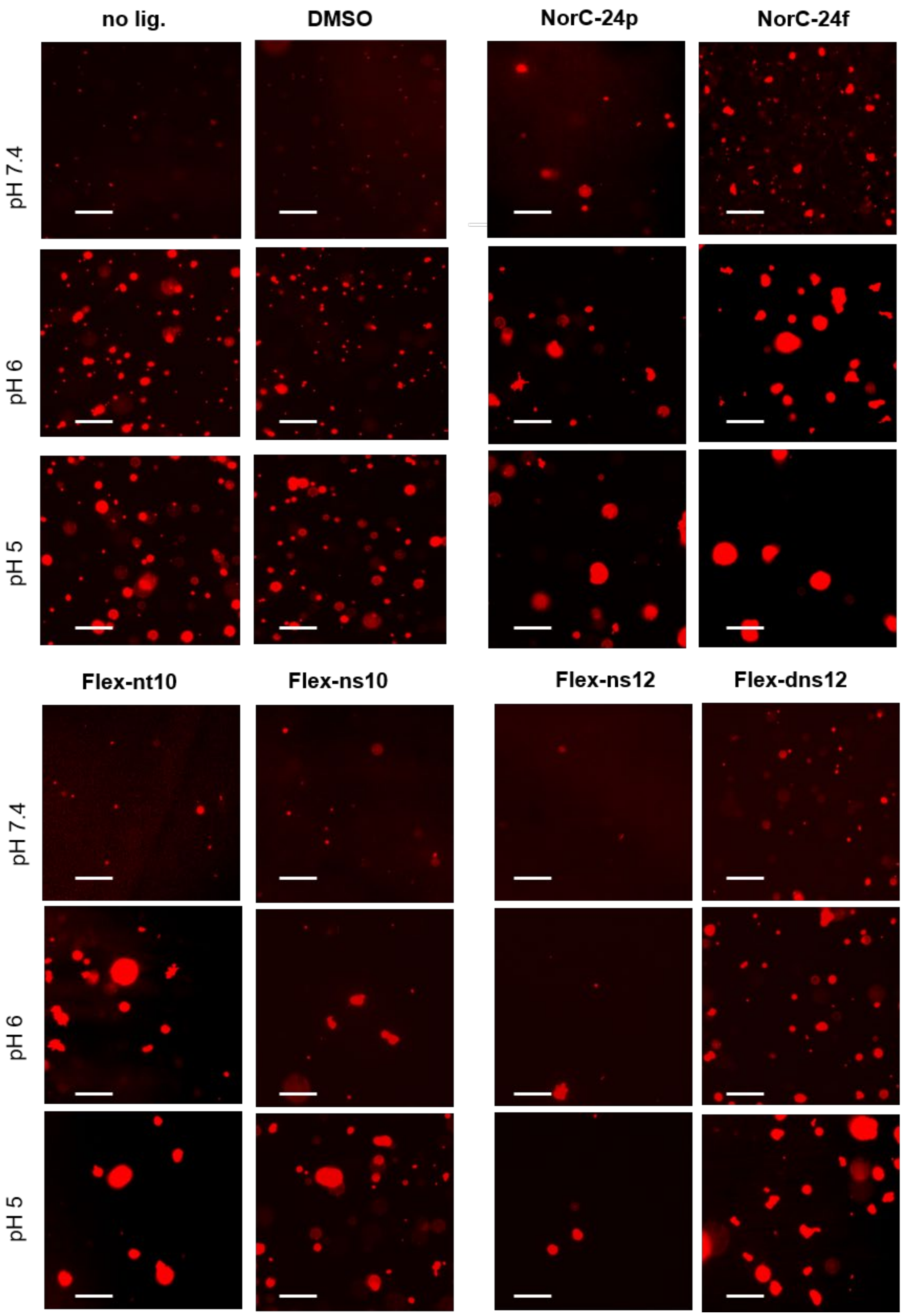

Figure S3. LLPS modulation assays with 5'-norcarbocyclic and fleximer nucleoside derivatives. Fluorescence microscopy images of mixtures of RED-labeled N (3 µM) and SL5_long (6 µM) were obtained after overnight incubation with 20 µM small molecule in the working buffers of different pH at 37°C. Scale bars: 10 µm.

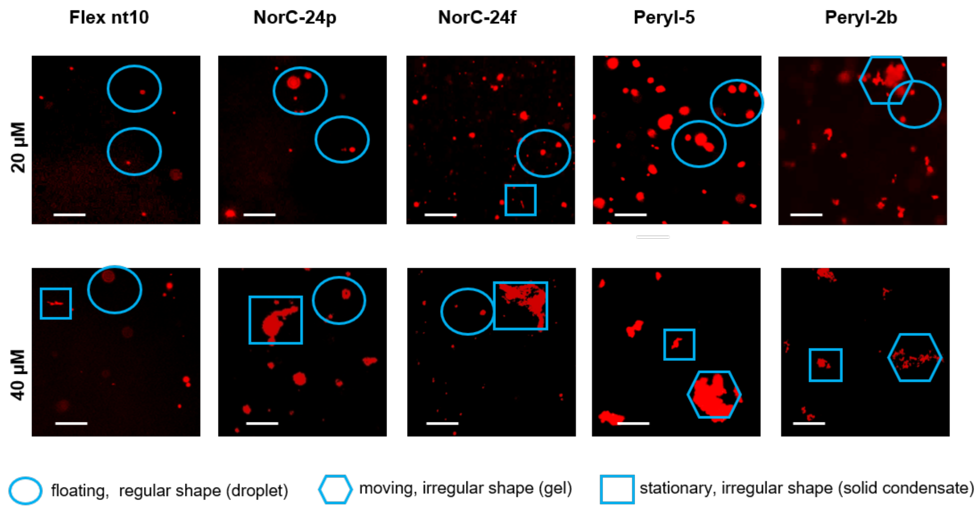

Figure S4. LLPS and other phase transitions at high concentrations of the small molecules. Fluorescence microscopy images of the mixtures of RED-labeled N (3 µM) and SL5_long (6 µM) were obtained after overnight incubation with 20 µM or 40 µM small molecule in the working buffer (pH 7.4) at 37°C. Scale bars: 10 µm. Marked areas were inspected visually for movement upon imaging to distinguish floating droplets and gels from solid aggregates.

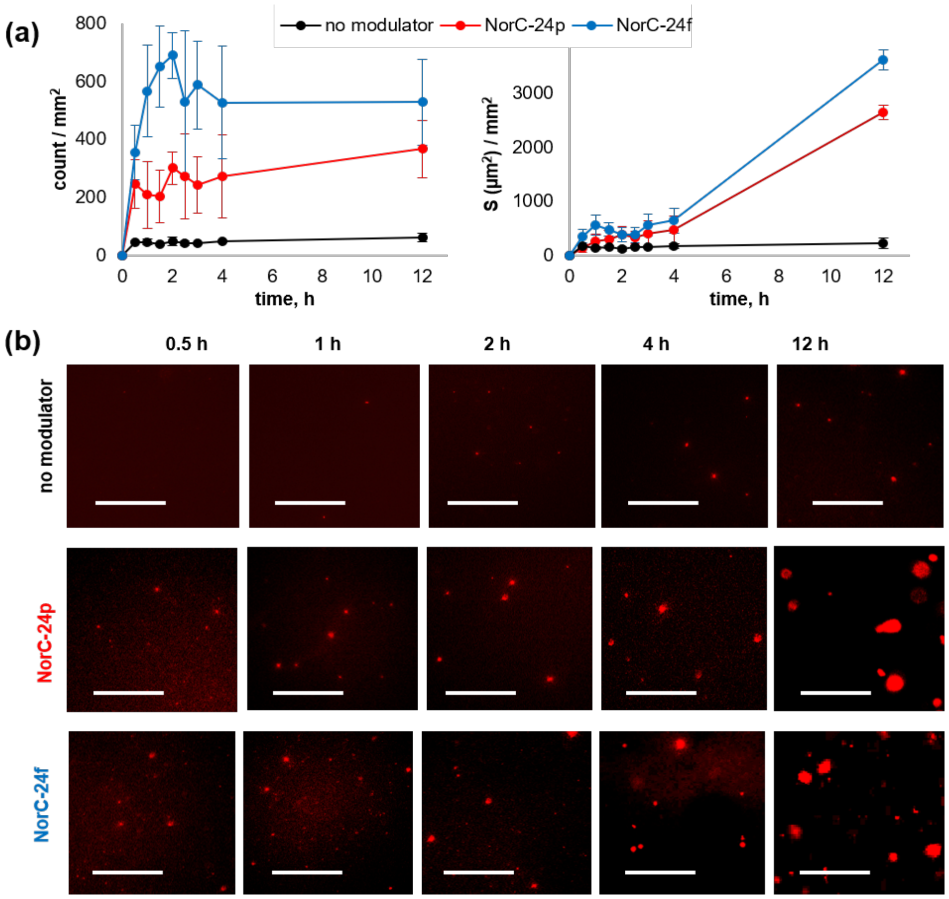

Figure S5. Droplet maturation assays with 5'-norcarbocyclic nucleoside derivatives. (a) Separation of RED-labeled N (3 µM) and SL5_long (6 µM) after 0.5-12 h of incubation with 20 µM NorC-24p/NorC-24f in the working buffer (pH 7.4) at 37°C. Average number of droplets (count) per mm^2 coverslip and their total area (S) were calculated based on fluorescence microscopy images. Error bars indicate standard deviation. All experiments were performed in triplicate. (b) Representative fluorescence microscopy images. Scale bars: 10 µm.

Text box S1. SARS-CoV-2 RdRp inhibition assay: *in vitro* transcription

We tested the ability of the ATP analog Flex-nt10 to inhibit SARS-CoV-2 RNA-dependent RNA polymerase (RdRp) *in vitro* using recombinant RdRp (the nsp12-nsp7-nsp8 complex) obtained as was previously described [1]. Two sets of synthetic RNA templates with respective γ-[P^{32}]-labeled primers were used in the assays:

1) the 50 nt template UUUAUCAAAAAGAGUAUUGACUUAAAGUCUAACCUAUAGGAUACUUACAG for incorporation of all 4 nucleotide triphosphates (NTPs) or their analogs; primer: GUUAGACUUUAAGUCAAU,
2) the 32 nt template 5'-UUUUUUUUUUAAUUCUUAAUCUCACAUAGC-3' for incorporation of ATP or its analogs; primer: GCUAUGUGAGAUUAAGAAUU.

The *in vitro* transcription assays were performed following a previously published protocol [1]. Briefly, RdRp (final concentration of 500 nM) was added to a preannealed primer-template solution (50 nM) in reaction buffer containing 10 mM Tris-HCl, pH 7.9, 10 mM KCl, 2 mM $MgCl_2$ and 1 mM DTT. The mixture was incubated for 15 min at 30°C, the reaction was initiated by adding NTPs at 30 °C and stopped with 1.1 volume of formamide with 100 $\mu g \cdot ml^{-1}$ heparin after 15 min. The reaction products were analyzed by 15% denaturing PAGE and visualized by phosphor imaging with a Typhoon 9500 scanner (GE Healthcare).

To analyze the effects of Flex-nt10 on RNA extension by RdRp, competitive assays with ATP were performed using both sets of templates-primers. In the case of template-primer 1, increasing concentrations of Flex-nt10 were added to the substrate-RdRp mixture along with ATP (3 µM or 10 µM final concentration) and other three NTPs (30 or 100 µM of each GTP, CTP, UTP). In the case of template-primer 2, only ATP was added. In both cases, RdRp successfully extended the primer to the end of template (Fig. S3a and S3b). In the case of template-primer 1, Flex-nt10 did not have significant effect on RNA extension (Fig. S3a). In the case of template-primer 2, Flex-nt10 inhibited primer extension with apparent EC50 ≈ 300 µM and 500 µM in the presence of 3 µM ATP and 10 µM ATP, respectively (Fig. S3b). Somewhat stronger inhibitory effect observed with the second template is likely explained by the presence of ten consecutive template uridines in its sequence, necessitating incorporation of multiple adenosine nucleotides during the reaction. Together, these results suggest that Flex-nt10 is not an efficient substrate for RdRp and cannot inhibit incorporation of ATP at physiological concentrations.

References

[1] N. Miropolskaya, M. Kozlov, I. Petushkov, M. Prostova, D. Pupov, D. Esyunina, S. Kochetkov, A. Kulbachinskiy. Effects of natural polymorphisms in SARS-CoV-2 RNA-dependent RNA polymerase on its activity and sensitivity to inhibitors *in vitro*. Biochimie 2022 https://doi.org/10.1016/j.biochi.2022.10.007

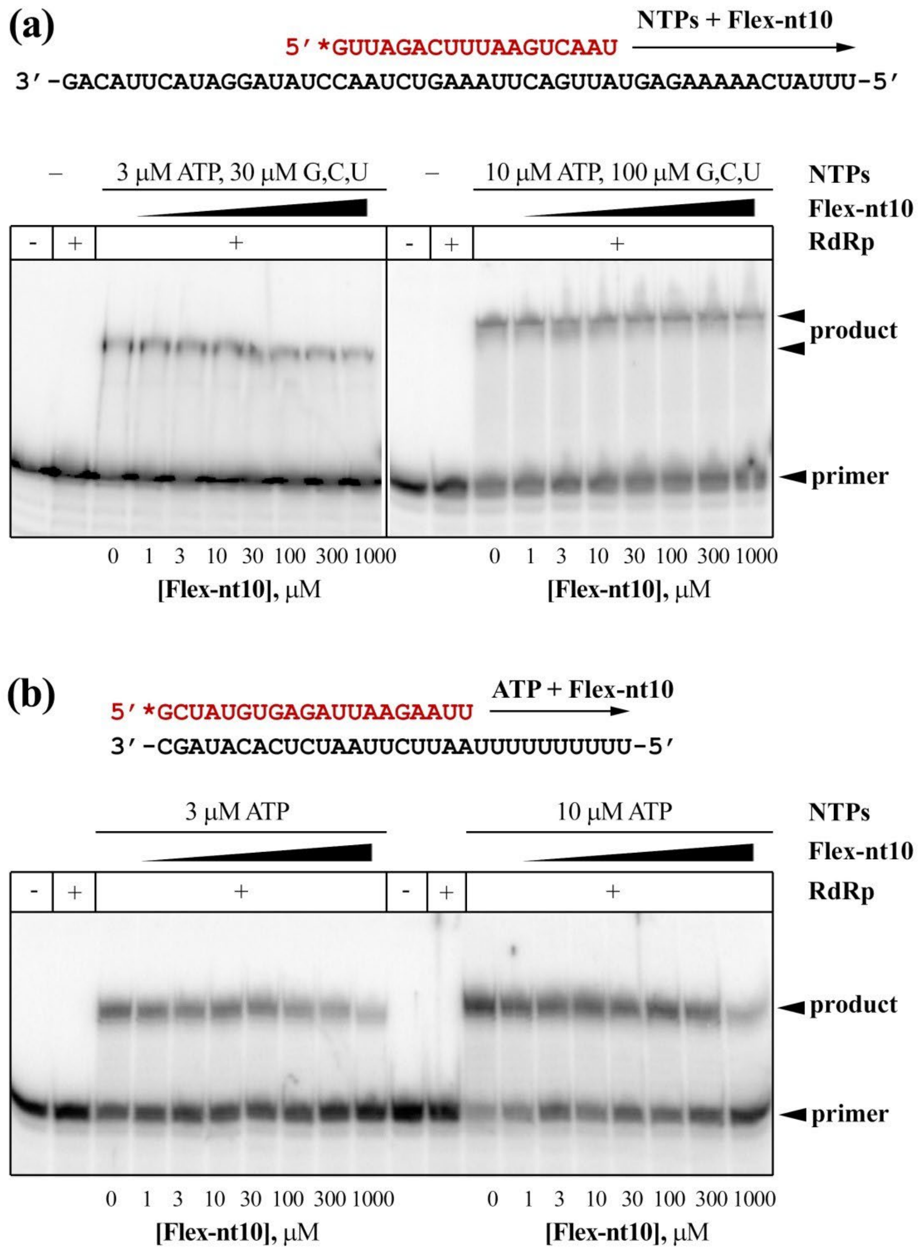

Figure S6. Inhibition of SARS-CoV-2 RdRp by Flex-nt10 in competitive assays. (a) PAGE analysis of primer extension in the complex of RdRp with template-primer 1 (50-nt template, mixed sequence) in the presence of NTPs and increasing concentrations of Flex-nt10. (b) Primer extension with template-primer 2 (32-nt template, oligo-U) in the presence of ATP and increasing concentrations of Flex-nt10.

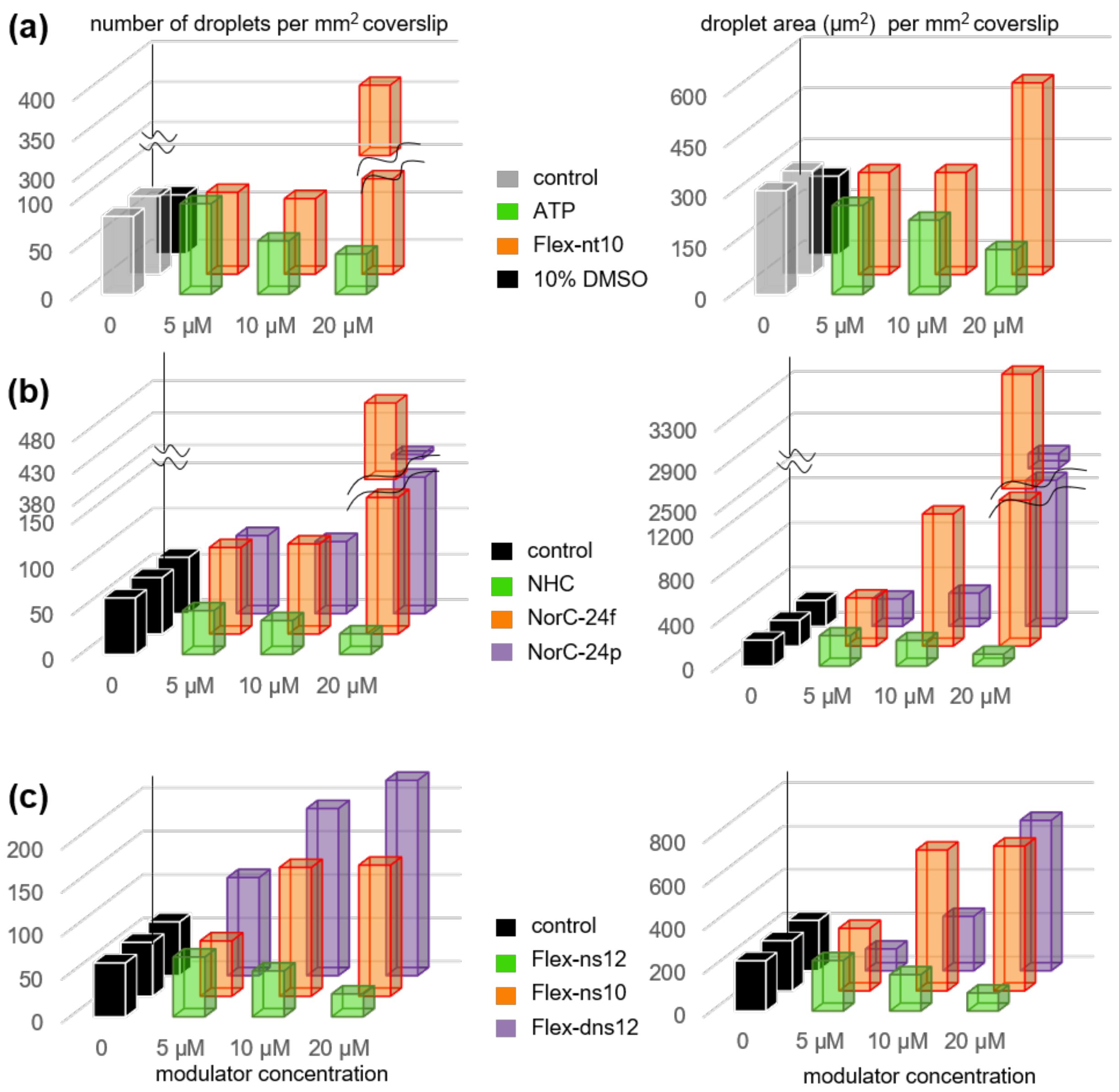

Figure S7. Concentration dependence of the effects of nucleoside analogs on N-RNA separation. Average number of droplets and the total droplet area were calculated based on fluorescent microscopy images obtained for the mixtures of RED-labeled N (3 µM) and SL5_long (6 µM) supplemented with 0-20 µM nucleoside analogs after overnight incubation at in 20 mM Tris-HCl buffer (working buffer, pH 7.4), at 37°C. (a) Phosphorylated purine nucleoside analogs; control: no ligand. (b) NHC and nor-carbocyclic pyrimidine nucleoside analogs (10% DMSCO); control: no ligand, 10% DMSO. (c) Fleximer purine nucleoside analogs (10% DMSCO); control: no ligand, 10% DMSO. All experiments were performed in three or more repeats, standard deviation was ≤ 18%.
